# The cGAS-STING pathway in viral infections: a promising link between inflammation, oxidative stress and autophagy

**DOI:** 10.3389/fimmu.2024.1352479

**Published:** 2024-02-15

**Authors:** Kunli Zhang, Qiuyan Huang, Xinming Li, Ziqiao Zhao, Chun Hong, Zeyi Sun, Bo Deng, Chunling Li, Jianfeng Zhang, Sutian Wang

**Affiliations:** ^1^ State Key Laboratory of Swine and Poultry Breeding Industry, Institute of Animal Health, Guangdong Academy of Agricultural Sciences, Key Laboratory of Livestock Disease Prevention of Guangdong Province, Scientific Observation and Experiment Station of Veterinary Drugs and Diagnostic Techniques of Guangdong Province, Ministry of Agriculture and Rural Affairs, Guangzhou, China; ^2^ State Key Laboratory of Swine and Poultry Breeding Industry, Guangdong Key Laboratory of Animal Breeding and Nutrition, Institute of Animal Science, Guangdong Academy of Agricultural Sciences, Guangzhou, China; ^3^ College of Animal Science and Technology, Guangxi University, Nanning, China; ^4^ Division of Nephrology, Shanghai Ninth People’s Hospital, Shanghai Jiao Tong University School of Medicine, Shanghai, China; ^5^ Maoming Branch, Guangdong Laboratory for Lingnan Modern Agriculture, Maoming, China

**Keywords:** cGAS-STING, viral infection, innate immune, autophagy, inflammation, oxidative stress, virus-host interaction

## Abstract

The host defence responses play vital roles in viral infection and are regulated by complex interactive networks. The host immune system recognizes viral pathogens through the interaction of pattern-recognition receptors (PRRs) with pathogen-associated molecular patterns (PAMPs). As a PRR mainly in the cytoplasm, cyclic GMP-AMP synthase (cGAS) senses and binds virus DNA and subsequently activates stimulator of interferon genes (STING) to trigger a series of intracellular signalling cascades to defend against invading pathogenic microorganisms. Integrated omic and functional analyses identify the cGAS-STING pathway regulating various host cellular responses and controlling viral infections. Aside from its most common function in regulating inflammation and type I interferon, a growing body of evidence suggests that the cGAS-STING signalling axis is closely associated with a series of cellular responses, such as oxidative stress, autophagy, and endoplasmic reticulum stress, which have major impacts on physiological homeostasis. Interestingly, these host cellular responses play dual roles in the regulation of the cGAS-STING signalling axis and the clearance of viruses. Here, we outline recent insights into cGAS-STING in regulating type I interferon, inflammation, oxidative stress, autophagy and endoplasmic reticulum stress and discuss their interactions with viral infections. A detailed understanding of the cGAS-STING-mediated potential antiviral effects contributes to revealing the pathogenesis of certain viruses and sheds light on effective solutions for antiviral therapy.

## 1 Introduction

The innate immune system is the first line of defence against viral infections. The initiation of this early immune response depends on the recognition of certain viral structures known as pathogen-associated molecular patterns (PAMPs). Hosts’ pattern recognition receptors (PRRs) recognize viral PAMPs, activating intracellular signalling pathways and inducing the expression of pro-inflammatory cytokines and antiviral genes that play antiviral effects. Through decades of research, six major classes of PRRs have been identified, including toll-like receptors (TLRs), retinoic acid-inducible gene (RIG)-I-like receptors (RLRs), NOD-like receptors (NLRs), C-type lectin receptors (CLRs), nucleic acid recognition receptors, and other innate immune receptors (such as scavenger receptors, complement receptors) (1, 2). These PRRs are mainly distributed on the cell surface, cytoplasm or lysosomes and induce innate immune responses and inflammatory responses through specific signal transduction pathways to promote virus clearance. In addition, the biological functions of PRRs have also included the activation of cells and complement, induction of cytophagy and cell death. Although the study of PRRs has been a hot area in immunology research, the role of these receptors in host defence and viral infection still needs to be further explored.

Interferon (IFN)-induced signalling pathway is the most important antiviral approach for the host and is activated by downstream signals of many PRRs (3, 4). Generally, binding of IFN to its receptor activates the downstream JAK-STAT pathway, resulting in increased transcription of IFN-stimulated genes (ISGs) (5). The ISG transcription proteins, such as myxovirus resistance (Mx), cholesterol 25-hydroxylase (CH25H) and oligoadenylate synthetase (OAS), play key roles in antiviral defences (6–8). In the early stages of viral infection, however, PRRs-mediated inflammatory response is also of great importance during antiviral processes. Interleukin-1 (IL-1) and tumour necrosis factor (TNF) can activate nuclear factor-κB (NF-κB) and induce IFN production, which further helps to remove viruses (9, 10). In addition, viral infection always affects cellular physiological states and metabolic processes, including oxidative stress, autophagy, and endoplasmic reticulum (ER) stress (11–13). Many studies have found that viral infections generally lead to a redox imbalance in the cellular environment (11). Oxidative stress is initially recognized as a means of combating viruses and protecting the host, contributing to apoptosis (14). However, with the development of research, more and more researchers found that oxidative stress promoted viral replication, which was a common mechanism used by some specific viruses (15). It is important to investigate the key molecular mechanisms used by viruses to interact with mitochondria and induce oxidative stress. As viruses need to use host cells to synthesize viral proteins, ER stress is always activated during viral infections. Understanding the complex mechanism of ER stress in viral infection is an important step in developing effective antiviral strategies. As an intracellular basic metabolic process (also known as type II programmed cell death), autophagy protects cells from toxic protein accumulation, organelle dysfunction, and viral infection by decomposing and recycling superfluous or potentially dangerous cytosolic entities. However, autophagy is a double-edged sword during viral infection. Studies have shown that some viruses have acquired the ability to hijack and subvert autophagy for their benefit (13). To sum up, all these factors affect the antiviral ability of the host.

As a newly identified PRR, cyclic GMP-AMP synthase (cGAS) recognizes viral, endogenous mitochondrial and genomic DNA in the cytoplasm and plays an important role in innate antiviral immunity (16). The conformation of cGAS changes upon binding to DNA, producing cGAMP, which is detected by the stimulator of interferon genes (STING) at the endoplasmic reticulum (17). Ishikawa and Barber have identified STING as an endoplasmic reticulum protein that has IFN-induced function in response to viral and intracellular DNA stimulation (18). The activated STING translocates to the Golgi apparatus, where it recruits TANK-binding kinase 1 (TBK1) and interferon regulatory factor 3 (IRF3) to form a complex (19). TBK1 then induces phosphorylation and oligomerization of IRF3. As a result, the activated IRF3 translocates into the nucleus, where it triggers the transcription of type I IFNs and ISGs that perform antiviral functions. Moreover, the cGAS-STING pathway is also involved in regulating the NF-κB-driven inflammatory immune response in vertebrate cells (20, 21). In addition, it has also been suggested that the cGAS-STING signalling axis is closely associated with oxidative stress, autophagy, and ER stress which affect the antiviral capability of the host (22–24). The inactivated STING is located in the endoplasmic reticulum, and the migration of activated STING is always accompanied by ER stress (25). Furthermore, ER stress can induce reactive oxygen species (ROS), which in turn, initiates the apoptotic process via constant oxidative stress (26). Additionally, the latest evidence suggests that the induction of autophagy is a highly conserved function of the cGAS-STING signalling axis (24). These researches suggest that these host cellular responses play significant roles in cGAS-STING-mediated viral infection. In this review, to further understand the regulatory mechanism among the cGAS-STING pathway, inflammation, IFN, oxidative stress, ER stress, and autophagy during viral infection, we discuss their interactions, which would facilitate revealing the pathogenesis of certain viruses and shed light on effective solutions for antiviral therapy.

## 2 Integrated omic and functional analyses identify the cGAS-STING pathway controlling viral infections and regulating various host defence responses

More and more multi-omics studies have confirmed the important role of cGAS-STING in the course of viral infections. Transcriptome analysis revealed that the expressions of IFNs (IFNA2, IFNA4, IFNA1, IFNA13, IFNB1, IFNL2 and IFNL3), ISGs (IFIT2, BST2, IRF7, OASL, MX1, IFITM1, IFIT2, IFI35, IFIH1, ISG15, CXCL10 and CXCL9) and pro-inflammatory cytokines (TNF, IL6, IL1B and IL1A) in skin from COVID-19 patients are significantly different from those of healthy donors (27). Further study found the activation of the cGAS-STING signal was the main cause of this large amount of type I IFNs and pro-inflammatory cytokines. In addition, cell death induced by severe acute respiratory syndrome coronavirus 2 (SARS-CoV-2) infection was also attributed to cGAS-STING activity. A proteomic study revealed that many vital PRRs, including TLR2, RIG-I, MDA5 (melanoma differentiation-associated gene 5) and cGAS, were upregulated in Japanese encephalitis virus (JEV)-infected fibroblasts (28). Similar results are also reported in SARS-COV-2, Zika virus (ZIKV), and dengue virus (DENV) infection (29–31). By analysing the mass spectrometry-based proteomic characterization of post-translational modifications, many novel sites of cGAS were identified, which affected cGAS activity and signal transduction (32–34). Recently, an interesting study showed that the gut microbiota can mediate peripheral cGAS-STING activation, which promotes host resistance to systemic viral infections (35). This evidence shows that cGAS-STING plays a key role in host resistance to viral infection. Further functional studies revealed that the activated cGAS phosphorylates its downstream effector protein STING at the Ser365 position upon viral infection and subsequently promotes type I IFN production and ISGs expression via TBK1-IRF3 and JAK-STAT pathways. These factors and signals are generally considered the most effective antiviral approaches (16, 36). Depletion of cGAS and STING enhanced virus replication and spread, which further confirmed their antiviral roles (28, 37). Although cGAS and STING play pivotal roles in the recognition of viral DNA, more and more evidence indicates they also play crucial functions in the host’s innate immune response against specific RNA viruses lacking DNA intermediates (38). Mice with a cGAS deficiency displayed heightened susceptibility to West Nile virus (WNV), a positive sense single-stranded RNA virus (36). The absence of cGAS likely results in a reduction of basal transcript levels of specific antiviral genes, making cells more susceptible to WNV infection. Simultaneously, mice lacking STING exhibit heightened susceptibility to RNA viral infections, and STING-deficient cells manifest an impaired ability to mount innate immune responses against RNA viruses, including vesicular stomatitis virus (VSV) and Sendai virus (SeV) (39). During RNA viral infection, it was observed that the cGAS-STING pathway is activated via indirect mechanisms, including the induction of mitochondrial stress and chromatin/nuclear membrane damage. This ultimately culminates in the liberation of intracellular double-stranded DNA into the cytoplasm, subsequently recognized by cGAS or alternative DNA sensors. RNA virus-induced cell membrane fusion has emerged as a pivotal process linking viral entry to the activation of STING. The comprehension of RNA viruses-cGAS-STING signalling interactions has markedly advanced, yet the precise mechanisms of activation of this pathway after RNA virus infections remain uncertain.

## 3 cGAS-STING-mediated IFN response is the crucial step in antiviral infection

cGAS is a cytosolic DNA sensor identified by Chen’s group in 2013 (16). It has been demonstrated that dsDNA activates cGAS in a length-dependent but sequence-independent manner (40). The dsDNA from various sources such as DNA viruses, retroviruses, bacteria, phagocytosed dead cells, and self-DNA leaked from damaged mitochondria could interact with cGAS. cGAS senses dsDNA and catalyses the production of cGAMP to bind the C-terminal domain (CTD) domain of STING and then changes the conformation of STING to oligomerize. The oligomerization of STING migrates away from the ER and activates TBK1 by phosphorylation at serine 365. The activated TBK1 then phosphorylates the CTT pLxIS motif (Ser366) of STING to recruit IRF3. TBK1 phosphorylates IRF3 and induces the IRF3 dimer to enter the nucleus, promoting type I IFN production. Activated IFN can lead to the up-regulation of several hundreds of ISGs, which in turn promotes the secretion of pro-inflammatory cytokines.

### 3.1 DNA/RNA viruses sensing by the cGAS-STING pathway

There have been sufficient reports on the recognition of DNA viruses by cGAS-STING signal. It has been demonstrated that cGAS-STING induces type I IFN production and further inhibits cytomegalovirus (CMV) replication in primary human endothelial cells (41). In the central nervous system, the activation of the cGAS-STING pathway suppresses herpes simplex virus 1 (HSV-1) replication in mice microglial cells (42). Moreover, the replication of hepatitis B virus (HBV) is inhibited due to activation of the cGAS-STING pathway in both human liver cell lines and *in vivo* mouse models (43). Another study also found that high-level expression of STING restricts susceptibility to HBV by mediating type III IFN induction (44). African swine fever virus (ASFV) is a complex, cytoplasmic double-stranded DNA (dsDNA) virus currently expanding worldwide. The cGAS-STING pathway is efficiently activated during NH/P68 attenuated strain infection, producing large amounts of IFN-β to inhibit ASFV replication. In contrast, the virulent Armenia/07 virus blocks the synthesis of IFN-β by impairing STING activation during infection (45). However, with further research, cGAS-STING has also been confirmed to play an important role in the response to RNA virus infection. In 2013, Schoggins et al. used an ectopic expression system to verify that cGAS also widely inhibits several RNA viral infections (36). During the human immunodeficiency virus (HIV) infection, cGAS senses its RNA-DNA hybrid and dsDNA, inducing IFN production to inhibit virus replication via the cGAS-STING pathway (46). During the SARS-CoV-2 infection, virus spike (S) protein induced cell fusion and then damaged nuclei to form micronuclei. The micronuclei are sensed by cGAS and lead to the activation of STING, which further induces type I IFN production (37).

### 3.2 Viruses inhibit cGAS-STING-mediated IFN production and antiviral function

As induction of type I IFN mediated by the cGAS-STING axis is crucial for host antiviral responses, viruses have evolved various strategies to antagonize this signalling pathway for immune evasion (**Table 1**). Numerous evasion mechanisms and immunomodulators have been identified in DNA viruses that target cGAS-STING signalling. It has been found that herpesviruses employed multiple strategies to antagonize the cGAS-STING pathway for immune evasion. The herpesvirus family includes HSV, CMV, varicella zoster virus (VZV), human herpesvirus (HHV), and Epstein Barr Virus (EBV), which are all DNA viruses. Human CMV (HCMV) tegument protein UL82 was reported to impair the translocation of STING from the ER to perinuclear microsomes and inhibit the recruitment of TBK1 and IRF3 to STING (58). Moreover, HCMV US9 was confirmed to disrupt STING oligomerization and STING-TBK1 association and block IRF3 nuclear translocation (59). The HSV-1 protein ICP27 interacts with the STING-TBK1 complex to inhibit IRF3 phosphorylation (53). The tegument proteins UL41 and UL46 of HSV-1 directly degrade cGAS mRNA or inhibit TBK1 activation, respectively (54, 55). Similarly, the murine CMV (MCMV) protein m152 was able to prevent the trafficking of STING from the ER to the endoplasmic reticulum-Golgi intermediate compartment (ERGIC), therefore inhibiting the interaction between STING and TBK1 (56). Pseudorabies virus (PRV) belongs to the alphaherpesvirus subfamily, which is also known as suid herpesvirus 1 or Aujeszky’s disease virus and infects a broad range of vertebrates. A recent study showed that PRV tegument protein UL13 functions as a suppressor of STING-mediated signalling to inhibit IFN production and antiviral response via recruitment of E3 ligase RING-finger protein 5 (RNF5) to induce K27-/K29-linked ubiquitination and degradation of STING (57). ASFV also uses different viral proteins to target the cGAS-STING pathway, inhibiting IFN production and escaping the innate immunity of the host. So far, it has been found that MGF360-15R (pA276R), pDP96R, pE120R, pI215L, pMGF505-7R and L83L protein encoded by ASFV target different adaptor proteins of the cGAS-STING pathway to inhibit type I IFN production (49, 60). In conclusion, maintaining high levels of IFN by ensuring the cGAS-STING activity is critical for host resistance to viral infection. Although cGAS-STING is considered the most potent signalling pathway to induce IFN, Kiran et al. found that JEV-induced type I IFN is cGAS-STING-independent (28). Most researchers believe that TLR and RLR are the main factors that induce IFN production. Interestingly, increased viral load was observed in a cGAS-depleted environment when IFN-β levels were still high. It suggested that the abundance of IFN-β transcripts was not sufficient alone to restrict viral replication. Therefore, there might be additional antiviral approaches regulated by the cGAS-STING signal. With the deepening of research, multiple functional roles and specific mechanisms of cGAS-STING during viral infections were identified, especially its effects on inflammation, oxidative stress and cell death.

**Table 1:** The interaction between virus and cGAS-STING pathway on type I IFN production.

Viruses	Target	Function	Reference
HIV	cGAS	Sensing RNA-DNA hybrid and dsDNA to induce IFN	(46)
SARS-CoV-2	cGAS	Sensing micronuclei to induce IFN	(37)
CMV	cGAS-STING-IRF3	The IFN-I response is dependent on cGAS-STING-IRF3 signalling	(41)
HBV	cGAS/STING	Activating the cGAS-STING axis to induce ISG56	(47, 48)
ASFV	cGAS/STING /TBK1/IRF3	Virulent factors target adaptor proteins of the cGAS-STING pathway to inhibit type I IFN.	(45, 49–52)
HSV-1	cGAS/STING/TBK1;	ICP27 interacts with STING-TBK1 complex to inhibit IRF3 phosphorylation; UL41 and UL46 degrade cGAS mRNA or inhibit TBK1 activity	(53–55)
MCMV	STING	M152 prevents the trafficking of STING from the ER to the ERGIC to inhibit the interaction between STING and TBK1	(56)
PRV	STING	UL13 recruits E3 ligase RNF5 to induce K27-/K29-linked ubiquitination, and STING degradation inhibit IFN	(57)

## 4 Function of cGAS-STING in regulating inflammation during viral infection

The host inflammatory response responds to harmful stimuli and is tightly regulated. After the PRRs recognize the invading virus, hosts initiate inflammatory signal transduction and trigger inflammatory responses, which play essential roles in early antiviral processes. The inflammatory response regulatory network plays a key role in the host antiviral process to maintain the body’s balance.

### 4.1 NF-κB is the key signal for cGAS-STING-induced inflammatory responses in viral infections

Recognition of viruses by PRRs causes the interaction of many adaptor molecules, which in turn initiate inflammatory signalling, including the NF-κB pathway, the JAK-STAT pathway, and the inflammasome pathway. The NF-κB pathway is thought to be the regulatory centre of the inflammatory response process. The NF-κB signalling pathway is involved in a variety of stress responses during viral infection, which in turn mediates various transcriptional processes and ultimately induces pro-inflammatory cytokine production. The SARS-CoV-2 infection causes varying degrees of respiratory symptoms and results in lung damage or even death in a significant number of cases. These severe cases are associated with high levels of pro-inflammatory cytokines and low antiviral responses (61). A recent study reported that in SARS-CoV-2 infected cells, the TBK1 and IRF3 pathways are blocked by several viral proteins. The SARS-CoV-2 infection causes mitochondrial stress/damage, DNA damage, cell death and leakage of mitochondrial DNA. These DNA activate the cGAS-STING axis and induce NF-κB activation to drive inflammatory immune response (21). cGAS-STING is recognized as a potential target for the treatment of SARS-CoV-2. And several STING-targeting drugs can attenuate the inflammatory response. The HIV/SIV (Simian immunodeficiency virus) research study showed that its Vpx proteins efficiently inhibit cGAS–STING-induced NF-κB signalling but not IRF3 activation, which further induces the production of several pro-inflammatory cytokines (62). In addition. ASFV protein pD345L has been found to suppress cGAS/STING-induced NF-κB activation (63). It is well known that NF-κB is the predominant regulator of inflammation and cGAS-STING can drive NF-κB activity during viral infections (21). Therefore, the role of cGAS-STING signalling in mediating inflammatory responses deserves more attention.

### 4.2 The cGAS-STING pathway interacts with the inflammasome complex in viral infections

NLRs also have powerful effects on inflammation induction. It has been proved that several NLRs, including NLRP1b, NLRP3, NLRC4, NLRP6 and NLRP12, are involved in the formation of inflammasome and regulate innate antiviral immunity. When viruses invade cells, NLRs recognize viral nucleic acids or endogenous molecules released from damaged or dying cells. Then, NLRs oligomerize and recruit pro-caspase-1 with or without ASC to form inflammasomes. In the inflammasome complex, caspase-1 can activate self-cleavage, and the activated caspase-1 cleaves pro-IL-1 and pro-IL-18 for their maturation and release. These mature pro-inflammatory cytokines then exert their antiviral function. IFN and pro-inflammatory cytokines are produced and function simultaneously during the host antiviral responses. Importantly, balance type I IFN production and inflammasome activation pathways are essential for immune homeostasis. Upon infection with HSV-1 or cytosolic DNA stimulation, STING engages with NLRP3, facilitating inflammasome activation via dual mechanisms (64). On one hand, STING recruits NLRP3 and promotes the localization of NLRP3 in the endoplasmic reticulum, thus promoting the formation of an inflammasome. On the other hand, STING interacts with NLRP3 to attenuate NLRP3 polyubiquitination associated with K48 and k63, thereby promoting inflammasome activation. It is widely known that the assembly of the NLRP3 inflammasome leads to the activation of caspase-1, which further results in the production of several pro-inflammatory cytokines. Caspase is the important link between inflammasome and inflammatory cytokines. Wang et al. found that caspase-1 interacted with cGAS to inhibit IFN production in DNA virus infection (65). This study also demonstrated that deficiency in inflammasome signalling enhanced host resistance to DNA viruses *in vitro* and *in vivo*. Moreover, this regulatory role also extended to other inflammatory caspases, including Caspase-4, 5, and 11 (65). These Caspases cut cGAS in conditions of non-canonical inflammasome activation. ZIKV, an RNA virus, has been reported to promote NLRP3 inflammasome activation to benefit its infection by stabilizing caspase-1 to suppress cGAS-mediated type I IFN signalling (31). The detailed mechanism is that the non-structural protein NS1 of ZIKV recruits the host deubiquitinase USP8 to cleave K11-linked poly-ubiquitin chains from caspase-1 at Lys134 to inhibit the proteasomal degradation of caspase-1. The enhanced stabilization of caspase-1 by NS1 promotes the cleavage of cGAS to inhibit the recognition of releasing mitochondrial DNA and then suppress type I IFN signalling. In addition, the activation of human caspase-3, an apoptotic caspase, has been demonstrated to cleave cGAS at D319, IRF3 at D121/125 and MAVS at D429/490, thus making apoptotic cells immunologically silent and negatively regulating DNA or RNA virus-induced cytokine production (66). Currently, there are few studies on the interaction between cGAS-STING and inflammasome signalling in viral infection, but the available evidence already suggests that the interplay between the cGAS-STING pathway and inflammasome complex affects IFN, inflammation and cell death. Therefore, this aspect deserves more attention.

## 5 The crosstalk between cGAS-STING signal and oxidative stress in viral infections

### 5.1 Oxidative stress is a double-edged sword in viral infections

Oxidative stress is an important pathological factor causing tissue damage, aging, tumours, and cardiovascular diseases. Under normal circumstances, oxidation and antioxidation are maintained in a balanced state. The oxidative and antioxidant systems in the body are disordered when harmful substances stimulate the organism. Excessive production of highly reactive molecules such as ROS and reactive nitrogen species (RNS) leads to the inhibition of antioxidant capacity, which tilts the equilibrium toward oxidation, resulting in oxidative stress. Oxidative stress is always associated with viral infections. Viral infection-induced ROS generation triggers oxidative stress in the organism and mediates apoptosis, which in turn mediates ROS and causes extensive damage, aggravating the disease process (67). For example, oxidative stress is a major characteristic of asthma and chronic obstructive pulmonary disease (COPD), and rhinovirus infection can make their condition worse. Oxidative stress attenuated the antiviral capacity of bronchial epithelial cells in asthma and COPD patients. Furthermore, oxidative stressor H_2_O_2_ could down-regulate the expression of epithelial cellular PRR TLR3 and antioxidants (SOD1 and SOD2), which suggested that ROS might have reduced the host’s antiviral capacity and promoted viral infection (68). But in some other studies, to a certain degree, oxidative stress activates the antioxidant defence system and autophagy in the tissues and organs, which help to scavenge some of the ROS and induce stress defence (69). Oxidative stress-induced ROS can also activate autophagy and apoptosis through various specific mechanisms, which induce cell death and inhibit virus replication. Moreover, H_2_O_2_ has been confirmed to regulate autophagy by inhibiting the autophagy-related gene (ATG) 4, which affects the lipidation of light chain 3 (LC3) and the degradation of pathogens (70). In addition, Latent Membrane Protein 1, a major EBV protein, facilitates ROS production, causes DNA damage and induces autophagy initiation (71). These studies suggest oxidative stress affects viral infection by directly regulating viral survival or indirectly affecting virus infection via apoptosis and autophagy.

### 5.2 cGAS-STING is a potential target that links oxidative stress and viral infection

**Figure 1:**
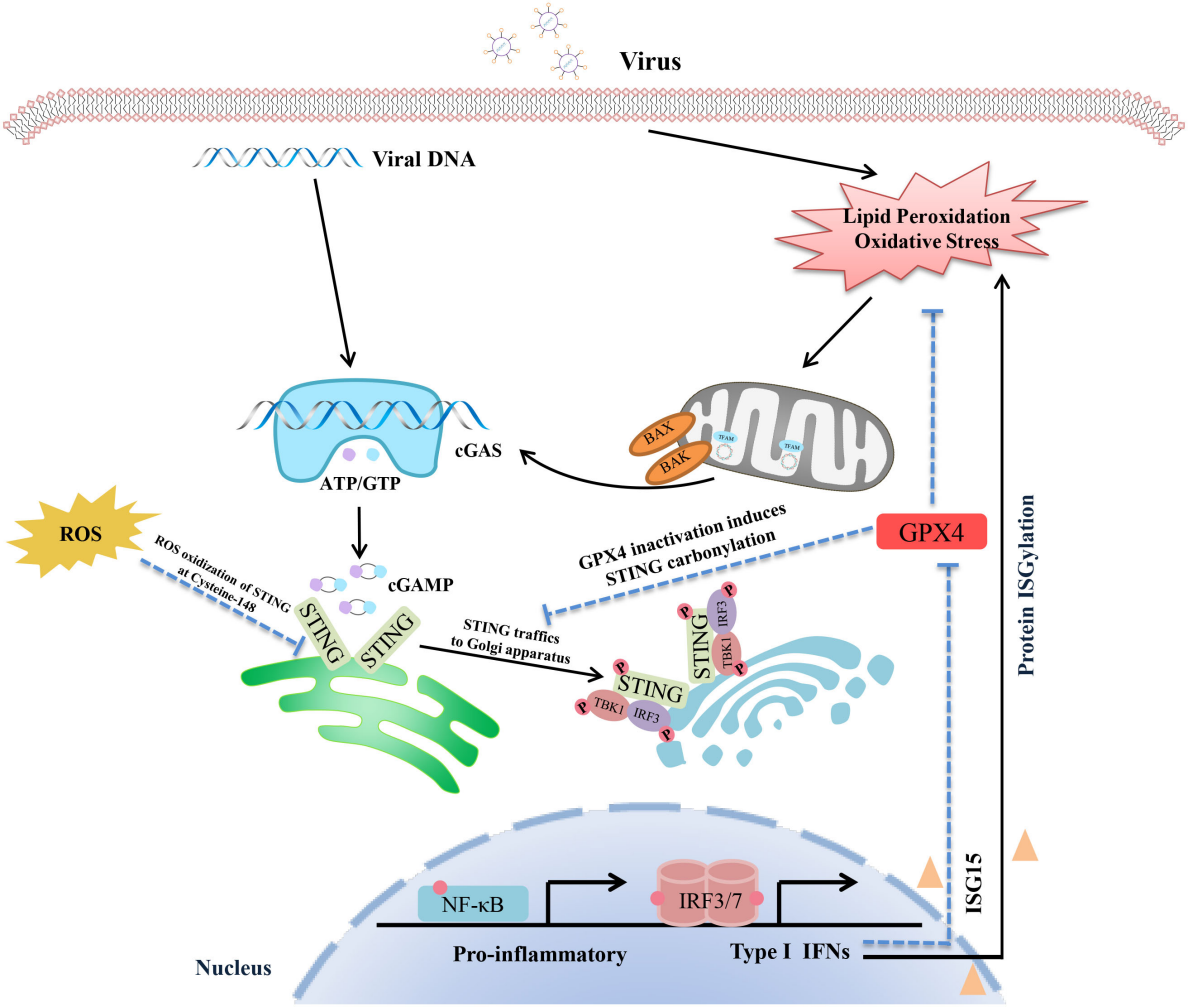
Schematic representation of the interaction between the cGAS-STING pathway and oxidative stress. The virus DNA and mtDNA can both be recognized by cGAS-STING signalling, inducing pro-inflammatory cytokines and IFN production via the TBK1-IRF3/NF-κB pathway. In addition, viral infection also triggers lipid peroxidation and oxidative stress, which lead to STING inactivation by facilitating STING carbonylation at C88. It is worth noting that GPX4 is a crucial nod connecting the cGAS-STING axis and oxidative stress. On the one hand, GPX4 activation inhibits oxidative stress, which ensures that the activated STING can be successfully transferred to the Golgi apparatus for further action. On the other hand, activation of the cGAS-STING-IFN axis promotes oxidative and inhibits GPX4 activity via ISG15 expression. In addition, ROS inhibits STING dimerization by oxidizing Cysteine 147 on STING. Redox modification of STING is an important regulatory mechanism of STING activity during viral infection.

It is widely known that the invading DNA virus will activate the cGAS-STING pathway, inducing type I IFN production and causing a range of innate immune responses. In recent studies, it has been found that STING is an upstream regulator of cellular oxidative stress. It is possible to regulate the level of lipid peroxidation and ROS by activating the cGAS-STING downstream signal ISG15. ISG15 is a member of the ISG family that induces IFN expression, contributes to “protein ISGylation”, and interferes with ubiquitin modifications. STING can negatively regulate the ubiquitin-proteasome system through ISG15, resulting in increased interferon-mediated ROS (72, 73). Indeed, IFN-mediated protein ISGylation regulates the ubiquitin-proteasome system to increase cellular ROS. Furthermore, glutathione peroxidase (GPX), an antioxidant molecule, attenuates oxidative stress by reducing H_2_O_2_ to water, which is also inhibited by ISG15. STING knockdown elevates glutathione peroxidase (GPX) activity via inhibition of ISG15. Recently, Hayman et al. also found the knockdown of STING down-regulated expression of ISG15 and ROS-related genes, including HECT domain and RCC1-Like Domain-Containing Protein 5 (HERC5), kruppel-like factor 4 (KLF4), and dual oxidase 2 (DUOX2) (73–76). These results suggest that STING is an upstream regulator of the intracellular oxidation processes. However, it is worth noting that some other studies believe that oxidative stress is an important inducement of cGAS-STING activation. During HSV-1 infection, GPX4 is indispensable for cGAS-STING activation. Actually, GPX4 inactivation leads to cellular lipid peroxidation, which decreases host innate antiviral immune responses and promotes virus replication via inhibition of the cGAS-STING signalling axis (22). Mechanistically, GPX4 inactivation did not affect the binding of viral DNA to cGAS but suppressed the trafficking of STING to the Golgi apparatus by facilitating STING carbonylation at C88. Another interesting study showed ROS promoted the replication of murine gammaherpesvirus-68 (MHV68), a close genetic relative of KSHV and EBV. ROS suppressed the production of IFN in a STING-dependent manner (77). ROS inhibits STING dimerization by oxidizing Cysteine 147 on murine STING during MHV68 infection. Redox modification of STING is an important regulatory mechanism of STING activity during viral infection. It is generally known that viral infection usually leads to oxidative stress in host cells, including SARS-COV-2, influenza virus and Hepatitis C virus (HCV) (15, 78–80). Oxidative stress is closely related to mitochondrial dysfunction, which triggers mitochondrial DNA (mtDNA) damage and DNA leakage, activating the cGAS-STING pathway (**Figure 1**) (81). It remains uncertain whether oxidative stress is the cause or the consequence of cGAS-STING signalling activation during viral infection. And whether oxidative stress induces STING activity or inhibits STING activation is also controversial. Meanwhile, there are few reports about the direct interaction between the cGAS-STING signal and oxidative intermediates. Therefore, more research is needed to explore their relationship. However, it must be admitted that the alteration of the levels of oxidative stress affects the cGAS-STING pathway and host antiviral immunity.

## 6 Function of cGAS-STING regulates autophagy during viral infection

### 6.1 Autophagy in antiviral host defences

To accommodate the diverse needs of metabolism, intracellular substances are constantly synthesized and degraded to maintain homeostasis. Autophagy is an evolutionarily conserved metabolic process of eukaryotic cells that degrades or recycles intracellular proteins and organelles and plays a key role in activating and regulating early immune responses during viral infection (82). PRR signals interact with autophagy adaptor proteins to regulate a series of immune responses, which effectively eliminates pathogenic microorganisms. For example, activation of the TLR-MYD88/TRIF pathway can disrupt the interaction between B cell lymphoma-2 (BCL-2) and Beclin-1, which induces autophagy (83). The recognition of VSV and SeV by TLR7 requires the transport of cytosolic viral replication intermediates into the lysosome. ATG5 deletion would reduce TLR7-mediated IFN production (84). Many studies have suggested that autophagy can degrade viral components, particles, and host factors, which functions as an effective innate antiviral mechanism. HCV non-structural 5A (NS5A) protein, which is crucial for HCV replication, can be degraded in autophagosomes. Autophagy helps to remove HCV in the presence of ER protein Scotin (85). Autophagy facilitates selectively degrading the HIV-1 transactivator Tat, inhibiting viral transcription and virion production in CD4^+^ T cells (86). There are also many other viruses, such as hepatitis B virus, porcine epidemic diarrhoea virus, and ZIKV, that are restricted by autophagy (87–89). On balance, viral infection is detected by multiple signalling pathways, and further triggers the activation of immune defences via autophagy.

### 6.2 Autophagy induction is an evolutionarily conserved function of the cGAS-STING signal

Earlier studies have mainly focused on the mechanism of IFN induction by the cGAS-STING pathway. This signalling pathway plays antiviral effects by regulating antiviral gene transcription. Of interest, a growing number of researchers find cGAS-STING signalling axis regulates virus clearance more immediately through autophagy. Of which, the main function of STING in combatting HSV-1 infection seems to be attributed to autophagy activation rather than type I IFN production (90). A study found a mouse model harbouring a serine 365-to-alanine (S365A) mutation in STING remained resistant to HSV-1, despite the loss of STING-induced IFN activity. It seems that the activation of autophagy, triggered by STING, is contingent upon CTT and TBK1, yet remains uninfluenced by IRF3. Therefore, understanding the molecular mechanism of autophagy regulation by the cGAS-STING pathway is crucial. Saitoh et al. first found that the dsDNA of pathogenic microorganisms could induce co-localization of STING, ATG9a and LC3, which are important autophagy proteins (91). Subsequently, STING was identified as an essential factor that triggered autophagy under the stimulation of microbial DNA, which could degrade pathogens by delivering them to autophagosomes (92). Furthermore, a study showed that cGAS could directly bind to the coiled-coil domain of Beclin-1, which is a pivotal protein for autophagy initiation (93). As a result, this interaction inhibits the synthesis of cGAMP and IFN and promotes autophagy-mediated cytosolic DNA degradation by releasing Rubicon from the Beclin-1 complex. Notably, Gui et al. explained the mechanism of STING-mediated autophagy without TBK1 activation and IFN induction (24). When pathogenic microorganisms infect cells, cGAS recognizes cytosolic DNA and synthesizes cGAMP, which further binds to STING. As a result, STING translocates to the ERGIC by interaction with SEC24C. Then, ERGIC acts as a membrane source for LC3 lipidation, promoting autophagosome formation that degrades the DNA virus. In many invertebrates, such as drosophila and sea anemone *Nematostella vectensis*, their STING only participates in autophagy induction but not IFN response (24, 94). These research suggest that autophagy induction is an evolutionarily conserved function of the cGAS-STING signalling axis which predates the emergence of the IFN signalling. Additionally, the structural analysis showed that STING had a conserved LIR domain which was exposed to the cytoplasm by conformational changes upon activation (95). Consequently, the exposed LIR domain could directly interact with LC3 to activate autophagy, leading to the degradation of STING itself and p-TBK1. This finding also showed STING could directly link immune activation to autophagy. So quite a few researchers believe that autophagy induction via STING trafficking is a primordial function of the cGAS pathway, which has long been thought that its primary function is to induce type I IFN production. Further studies show that STING orchestrates endoplasmic reticulum stress and the unfolded protein response via a novel UPR motif within the cyclic-dinucleotide-binding (CBD) domain. This motif exerts a negative regulatory effect on the Akt/tuberous sclerosis complex (TSC)/mammalian target of the rapamycin (mTOR) pathway, thereby amplifying canonical autophagy (25, 96). Several other studies also revealed the mechanisms of STING-mediated noncanonical autophagy (97, 98). Activated STING translocates from the endoplasmic reticulum to the ER-Golgi intermediate compartment and Golgi apparatus, contingent upon the coat protein II (COP II) complex and Arf GTPases. The ERGIC serves as a membrane reservoir for LC3 lipidation and the genesis of autophagosomes. Different from canonical autophagy, STING-elicited noncanonical autophagy operates independently of upstream autophagy modulators, including unc-51-like kinase 1 (ULK1), Beclin-1, and ATG9a, yet relies on downstream autophagy regulators such as ATG5 and ATG16L1 (24).

### 6.3 Viruses evade host immune defence by inducing autophagic degradation of cGAS/STING

**Figure 2:**
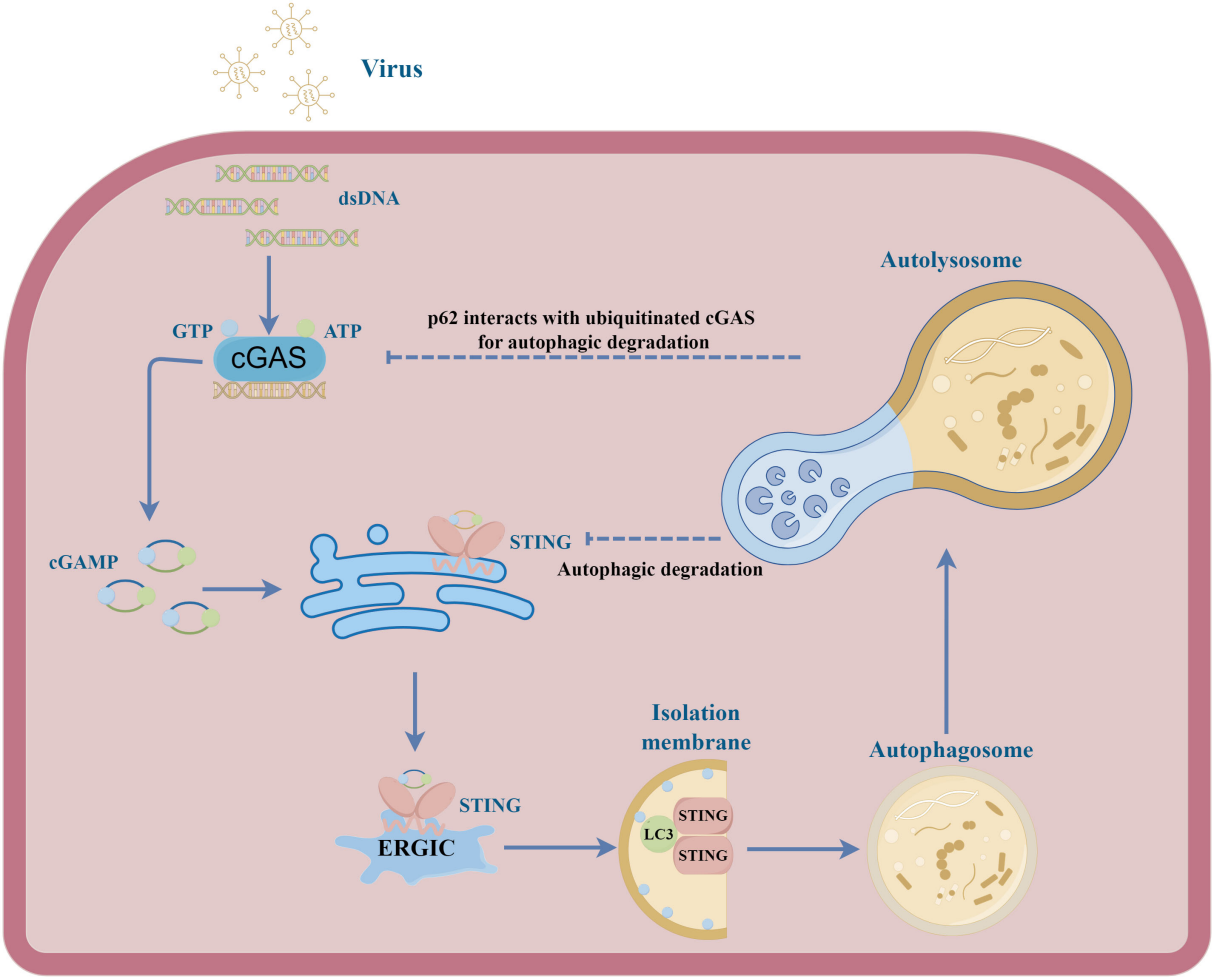
A schematic illustration depicting the interplay between cGAS-STING signal and autophagy in viral infection. Upon activation by cGAMP, STING undergoes translocation from the endoplasmic reticulum to the ERGIC. Within the ERGIC, STING has been implicated in the initiation of autophagy. The STING-containing ERGIC functions as a membrane source of LC3 lipidation, thereby triggering the formation of the autophagosome. Ultimately, the autophagosome fuses with the lysosome, effectuating the degradation of its contents. During different viral infections, the cGAS and STING can be degraded in autolysosome, inhibiting host antiviral responses.

Viruses have also evolved unique mechanisms to ensure their survival by influencing autophagy processes and cGAS-STING signalling. ASFV MGF505-7R, MGF505-11R and L83L proteins promote autophagy-lysosomal degradation of STING, thereby blocking the phosphorylation of the downstream signalling molecules TBK1 and IRF3 and impairing type I IFN production (51, 60, 99). PCV2 infection can induce cGAS degradation via the autophagy-lysosome pathway (100). Mechanically, PCV2 infection triggers the phosphorylation of cGAS at S278 through the PI3K/Akt pathway. This phosphorylation of cGAS promotes the K48-linked poly-ubiquitination of cGAS which interacts with autophagy receptor p62 for autophagic degradation in autolysosome. As a result, the autophagic degradation of cGAS inhibits cGAMP and IFN-β production, which further impair hosts’ innate antiviral responses. Similarly, HBV X protein also can inhibit type I IFN production by boosting ubiquitination and autophagic degradation of cGAS (101). Except for cGAS, another autophagy receptor Coiled-coil domain containing 50 (CCDC50) associates with and targets STING for autophagic degradation (102). The MIU motifs of CCDC50 can recognize K63-polyubiquitinated STING and facilitate the conveyance TBof K63-polyubiquitinated STING to LC3B-marked autophagosomes, subsequently initiating autophagic degradation in a p62-independent way. During HSV-1 infection, the absence of CCDC50 promotes IFN and pro-inflammatory cytokines production and inhibits HSV-1 replication. These results suggest the autophagic degradation of cGAS-STING signalling during infections has a significant impact on type I IFN production and viral replication (**Figure 2**).

### 6.4 cGAS-STING-induced autophagy not only exerts direct antiviral effects but also influences host antiviral responses by affecting IFN signalling

Although many studies have been conducted on viral evasion of STING-induced IFN-mediated antiviral function, investigations about viral evasion of STING-induced autophagy-mediated antiviral function remain notably limited. Recently, an interesting study found that the bat STING can only induce autophagy and antiviral activity but not IFN induction (103). SARS-CoV-2 ORF3a constitutes a distinctive viral protein capable of interacting with STING, consequently disrupting the STING-LC3 association and impeding cGAS-STING-mediated autophagy, whilst preserving IRF3-Type I IFN induction. This novel functionality of ORF3a, different from targeting autophagosome-lysosome fusion, is a selective impediment of STING-mediated autophagy, thereby promoting viral proliferation. In addition, the interaction between the TBK1-IRF3-IFN pathway downstream of cGAS-STING and autophagy in viral infections is very complex. During infection, excessive accumulation of STING will trigger a strong inflammatory reaction, leading to deleterious effects on the host (93, 104). When the cGAS-STING pathway is activated, TBK1-IRF3 signalling downstream of STING will phosphorylate p62 at S403, which has a remarkably high affinity for ubiquitinated STING. As a result, the ubiquitinated STING is degraded in autophagosomes in an IRF3‐dependent manner (105). Moreover, another research group also found that TBK1 could phosphorylate selective autophagy receptors optineurin (OPTN), NDP52, and TAX1BP1 linking ubiquitinated cargo to autophagic membranes (106). As is known to us all, type I IFN participates in activating JAK/STAT and PI3K/Akt pathways, which are always involved in autophagy induction (107). Type I IFN does not induce autophagy in STAT-deficient cells (108). PI3K/Akt signalling axis inhibits autophagy by activating mTORC1 and inhibiting the expression of forkhead box O (FOXO) and autophagy-related genes. At later time points, negative regulators of the PI3K/AKT/mTOR pathway are induced, inhibiting mTORC1 activity and inducing autophagy (107). Therefore, the TBK1‐IRF3-IFN axis plays a crucial role in activating and regulating the host’s immune and autophagy. At present, there are still some problems plaguing us. Some researchers believe cGAS-STING-mediated autophagy plays an antiviral role (90, 103, 109). But other research groups suggest that cGAS-STING-mediated autophagy contributes to inhibiting the antiviral function of the host by degrading cGAS/STING directly or by degrading key proteins downstream of the cGAS-STING pathway (**Table 2**) (51, 100, 117, 118). As a result, this process inhibits IFN production. The next question that needs to be solved is how to control the target of autophagic degradation in viral infection.

**Table 2:** cGAS-STING-mediated autophagy plays a dual role during viral infection.

Viruses	Target	Function	References
HSV-1	STING	An S365A mutation in STING is resistant to HSV-1 by activating autophagy, despite lacking IFN responses	(90, 110)
HSV-1	cGAS-Beclin-1	The direct interaction between cGAS and Beclin-1 enhances autophagy-mediated pathogen DNA degradation	(93, 111)
HSV-1	GBP1-STING	GBP1 combines with STING and promotes autophagy, inhibiting HSV -1 infection in an IFN-independent manner	(112)
ZIKV	NF-κB-STING	In invertebrates, ZIKV-dependent NF-kB activation induces antiviral autophagy via activation of STING	(87, 113, 114)
HRV	STING	The STING-mediated antiviral activity required the induction of ATG5-dependent autophagy	(115)
PPRV	STING	STING regulates PPRV replication by activating the ATF6 pathway of UPRs to induce autophagy	(116)
ASFV	STING	ASFV MGF505-7R/11R interacted with STING and degrades STING expression by autophagy pathways, facilitating virus proliferation	(51, 99)
ASFV	cGAS-STING-TBK1	ASFV pA137R negatively regulates the cGAS-STING-mediated IFN via the autophagy-mediated TBK1 degradation	(117)
ASFV	TBK1-IRF7	ASFV MGF360-11L interacted with TBK1 and IRF7, degrading TBK1 and IRF7 via autophagy pathways.	(118)
PCV2	cGAS	PCV2 induces the cGAS ubiquitination degradation by autophagy, promoting virus infection	(100)

## 7 Interactions between the cGAS-STING axis and ER stress during viral infection

### 7.1 The ER stress responses have important influences on viral survival

There is adequate evidence that the state of the endoplasmic reticulum also influences a variety of selective autophagy, including mitophagy and ER-phagy (119, 120). To further expand our understanding of the effects of cGAS-STING on autophagy and oxidative stress, we focus on the endoplasmic reticulum. The endoplasmic reticulum is a continuous membrane system widely distributed in the cytoplasm. It mainly performs the functions of intracellular material transport, glucose and lipid metabolism, and protein processing. In addition, ER also provides a membrane structure for the formation of autophagosomes and peroxisomes. Many viruses use the ER as a replication site, where they synthesize proteins, replicate genomes, and assemble virion (121). ER stress is usually triggered by calcium homeostasis disequilibrium, unfolded protein (UPR) accumulation and lipid dysregulation (122). ER stress is also considered to be a potential cause of mitophagy and ER-phagy (123, 124). Accumulation of viral proteins in ER can also induce ER stress (125). ER stress initiates UPR-mediated protein degradation pathways, apoptosis and autophagy in host cells, inhibiting or degrading the accumulation of viral proteins to maintain cellular homeostasis. Generally, different viruses selectively activate the PERK (proline-rich extensin-like receptor kinase)-eIF2α (eukaryotic translation initiation factor 2) pathway, IRE1α (inositol-requiring enzyme 1α)-XBP1 (X-box binding protein-1) pathway or ATF6 (activating transcription factor 6) pathway, leading to ER stress. Transmissible gastroenteritis virus (TGEV) can activate the PERK-eIF2α signalling pathway and subsequently diminish the synthesis of viral proteins by decreasing protein translation efficiency (126). The HCV negatively regulates ER stress via the IRE1α-XBP1 pathway, increasing the synthesis of viral proteins and facilitating viral infection (127). Influenza A virus promotes viral replication by inhibiting ER stress response factor XBP1 and limiting host protein production to alleviate ER stress (128). However, the UPR remains to be a double-edged sword during viral infection. Some viruses regulate UPR to promote survival by activating other cellular responses. For example, duck enteritis virus (DEV) can activate ER stress and autophagy in a PERK-eIF2α/IRE1α-XBP1 dependent manner. Inhibiting the expression of PERK and IRE1 helps to suppress autophagy and DEV replication (129).

### 7.2 Endoplasmic reticulum localization of STING underlies its interaction with endoplasmic reticulum stress signalling

During viral infection, the viral DNA can be recognized by the cGAS and activates the cGAS-STING pathway, triggering a series of immune and cellular responses to protect the body, including ER stress (23). Notably, the inactivated STING is located on the outer membrane of the ER, and the migration of the activated STING and the activation of the STING-TBK1-IRF3 signal always occur simultaneously with ER stress. Several recent studies have shown a partial overlap between ER stress signals and the cGAS-STING signalling axis. During pathogenic microbial infection, phosphorylation of PERK was significantly impaired in STING-deficient macrophages. STING gain-of-function mutant N154S induces chronic ER stress by disrupting Ca2+ homeostasis. A newly identified STING CTD motif is involved in mediating ER stress in an IFN-independent manner (96). Similarly, deletion of the Ca^2+^ sensor STIM1 leads to spontaneous activation of the STING-TBK1-IRF3 pathway, which results in type I IFN-mediated ER stress (130). Moreover, higher levels of PERK phosphorylation were induced at times of the expression of STING (25). Furthermore, co-immunoprecipitation assay suggests that STING and PERK can interact directly and promote the removal of pathogenic microorganisms. Additionally, down-regulating the expression of PERK or IRE-1 inhibits STING activity (131). These research imply a link between ER stress and cGAS-STING signal. The interrelationship between downstream signalling molecules of cGAS-STING and ER stress was also illustrated in several reports. The activation of IRF3 by STING is initiated by ER stress (132). The signal that triggers the phosphorylation of IRF3 is derived from the ER. ER stress triggered the phosphorylation of IRF3 at S386 in an XBP1-independent manner, promoting IRF3 nuclear translocation (133). Moreover, ER stress can mobilize the ER-resident STING and facilitate the co-localization of STING and TBK1. Another research found that XBP1 splicing and IRF3 phosphorylation depend on the presence of STING (132). There is also evidence suggesting that several genes, including tyrosine kinase 2 (TYK2), STAT2 and IRF9, take part in IFN-induced ER stress, but the specific mechanism is still unclear (134). As yet, studies on the interaction between ER stress and the cGAS-STING pathway are mainly focused on metabolic and autoimmune diseases. More research is needed to further understand their role in viral infections.

## 8 Summary and perspectives

**Figure 3:**
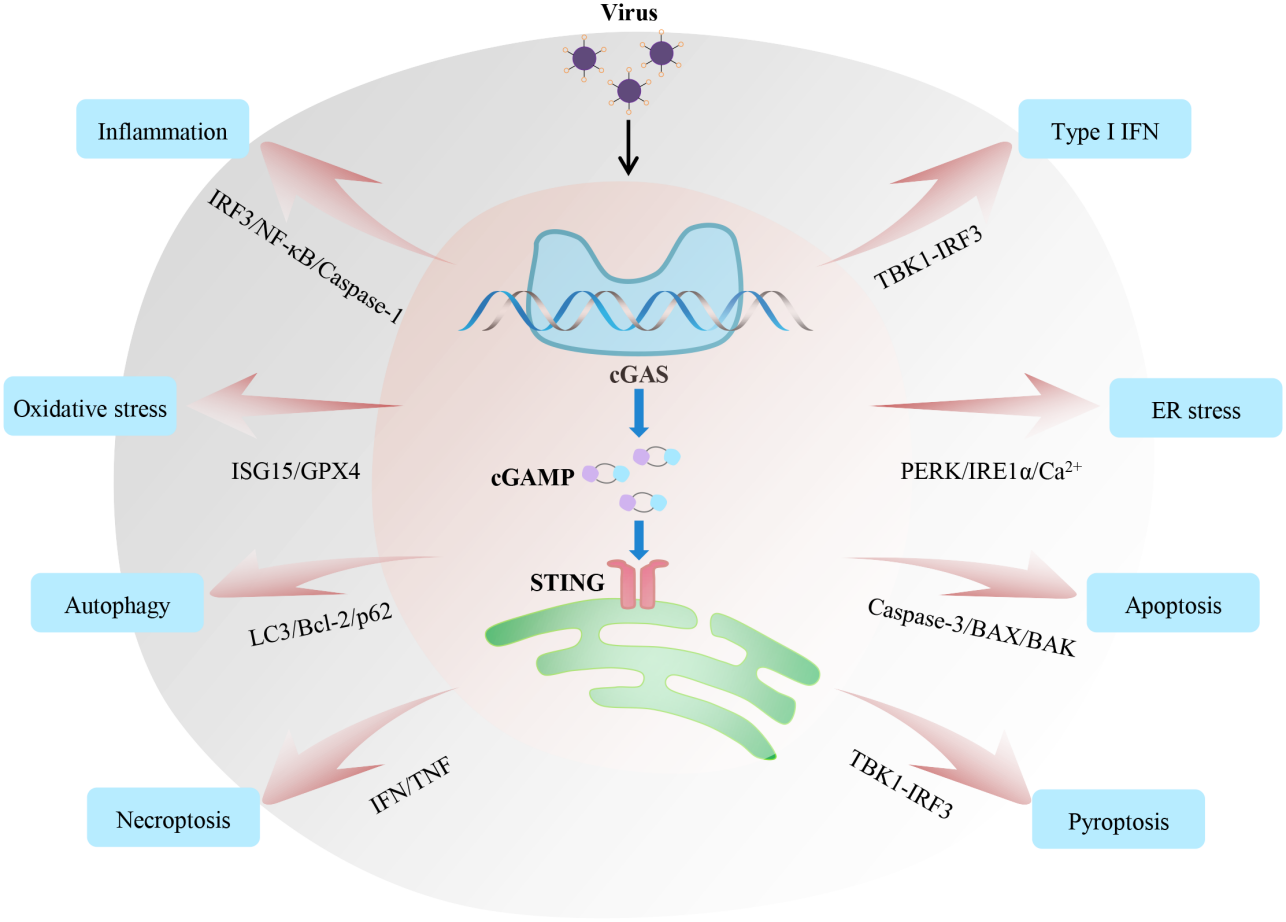
Regulatory mechanisms and functions of the cGAS-STING axis during viral infection. The cGAS-STING signalling axis widely participates in various immune and cellular responses, including inflammation, IFN, oxidative stress, endoplasmic reticulum stress, and different types of cell death during viral infection. All these responses affect the host’s ability to fight off invading viruses. Based on relevant studies, we summarize the crucial signalling nodes or proteins involved in these processes.

Viral infection and its serious consequences constantly threaten people’s health and safety. Therefore, understanding the molecular basis of host antiviral immunity is beneficial for eliminating viruses and attenuating physiological impairments. Recent research on the cGAS-STING pathway has increased our understanding of the recognition and removal of viruses. Although we have outlined recent insights of cGAS-STING in regulating IFN, inflammation, oxidative stress, autophagy and endoplasmic reticulum stress upon virus infections, this signalling axis is also involved in some other early host antiviral processes, such as different types of cell death and metabolism (**Figure 3**). Stimulation with a high concentration of HSV-I triggers cGAS-STING-dependent apoptosis, which affects local immune responses (135). Mechanistically, the activated cGAS-STING promotes the accumulation of phosphorylation of IRF3, which relieves the inhibitory effect of Bcl-xL on mitochondrial outer membrane permeability and further induces apoptosis. In addition, MHV68 leads to STING-dependent necroptosis in primary macrophages (136). Type I IFN works in coordination with TNF to induce necroptosis through STING activation. Moreover, mtDNA stress can activate the cGAS-STING-mediated DNA sensing pathway, inducing autophagy-dependent ferroptosis via lipid peroxidation (137). Also, several studies have shown that the activation of STING contributes to pyroptosis via the TBK1-IRF3 signal (138, 139). We speculate that inflammasomes and lysosomes may be the key links among different types of cell death downstream of cGAS-STING pathways during viral infection.

Notably, three different research groups have found that mtDNA released from mitochondria could activate the cGAS‐STING signalling axis (81, 140, 141). That is, the cytoplasmic cGAS-STING can recognize both the invading pathogenic DNA and endogenous DNA. mtDNA is a double-stranded, circular molecule, which can be recognized by TLR9, AIM2 and cGAS, inducing immune responses (142–145). West et al. found mitochondrial transcription factor A (TFAM, a key regulatory factor in mtDNA transcription and replication) deficiency and mitochondrial stress would cause the leakage of mtDNA into the cytoplasm, activating the cGAS-STING axis and initiating type I IFN response (81). HSV-1 and VSV infection can induce TFAM depletion and mitochondrial stress, facilitating mtDNA release into the cytoplasm and triggering cGAS-STING-mediated antiviral immune responses (146). Mitochondrial dysfunction is not only the result of oxidative stress and inflammatory responses but also a trigger for selective autophagy (mitophagy). Moreover, mitochondrial dysfunction-mediated mtDNA cytosolic leakage can trigger antiviral innate immune response by activating the cGAS-STING pathway. Therefore, we believe that the mitochondrial dysfunction events in viral infections are key to linking cGAS-STING signalling, inflammation, oxidative stress, and autophagy.

Interestingly, RNA viruses, such as SARS-CoV-2, HIV and DENV, also activate the cGAS-STING axis, despite cGAS being a DNA PRR (36, 46, 147). Mechanistically, the activation of the cGAS-STING by retroviruses depends on their reverse transcription to produce DNA. cGAS-STING activity induced by other RNA viruses is partly due to mitochondrial damage caused by a viral infection, which in turn leads to the accumulation of mtDNA in the cytoplasm (30). Therefore, during virus invasion, we should not only consider the activation effect of the virus itself on the cGAS-STING signal but also pay attention to the influence of cellular physiological changes on it.

Although the advent of omic technologies greatly expands the objectives of our study, each omics analysis still has some limitations for different samples. Meanwhile, the occurrence and development of viral diseases is a complex network, and many factors, such as gene mutation, abnormal transcription and epigenetic changes, affect the host’s physiological status. Combined multi-omics analysis can analyse multiple consecutive events of disease occurrence and identify the antiviral targets more precisely. Moreover, with the rapid development of gene-editing technology, using genome-wide CRISPR screening to identify host factors of the virus-infected cells is a current research hotspot. Integrating genome-wide CRISPR screening with multi-omic data seems to be a promising approach to understanding the virus-host interactive network. A research group have used this strategy to identify some novel and effective antiviral factors (148). This method may help develop new strategies for improving host disease resistance and antiviral therapy.

Indeed, the cGAS-STING pathway plays a dual role in early antiviral immunity and cellular responses. On the one hand, intracellular DNA induces various cellular responses and the expression of type I IFN and pro-inflammatory cytokines to fight against invading viruses via the cGAS-STING-TBK1-IRF3/NF-κB axis. On the other hand, the invoked cell death and intracellular stress responses can regulate the upstream regulators and downstream effectors of cGAS-STING, affecting immune responses and pathogen clearance. For example, cGAS-STING-activated autophagy, in turn, degrades STING and suppresses the immune response (105). Some viruses have evolved various strategies to antagonize the cGAS-STING pathway for immune evasion. Under different infectious conditions, the activations of cGAS-STING signalling are not the same. The inactivation and overactivation of the cGAS-STING signal are both detrimental to pathogen clearance by the host. Inhibition of the cGAS-STING axis suppresses host antiviral responses. And overactivation of cGAS-STING would trigger a strong inflammatory reaction and drive immunopathology. Of great concern, cGAS/STING has become an effective drug target. Researchers are working on designing or screening small molecule drugs that can regulate cGAS/STING activity. Presently, great progress has been made in the research of cGAS inhibitors. Some drugs can directly interfere with DNA binding to cGAS or competitively bind cGAS, thereby inhibiting the initial activation of cGAS (149). However, the agonists targeting cGAS are relatively rare and need further study. Although research on cGAS-STING has become increasingly mature, how to accurately regulate the cGAS-STING activity and promote virus elimination by host cells still needs further exploration.

## Author contributions

KZ: Conceptualization, Writing – original draft, Writing – review & editing. QH: Writing – original draft. XL: Writing – review & editing. ZZ: Writing – review & editing. CH: Writing – review & editing. ZS: Writing – review & editing. BD: Writing – review & editing. CL: Writing – review & editing. JZ: Conceptualization, Funding acquisition, Writing – review & editing. SW: Conceptualization, Writing – original draft, Writing – review & editing.
